# Management of Maxillary Incisors With Middle-Third Root Perforation: A Case Report

**DOI:** 10.1155/2024/5957016

**Published:** 2024-10-22

**Authors:** Daysi Morocho-Monteros, María José Masson-Palacios, Juan Marcos Parise-Vasco

**Affiliations:** ^1^Postgraduate Programme in Endodontics, Faculty of Health Sciences Eugenio Espejo, Universidad UTE, Quito, Ecuador; ^2^Centre for Research in Public Health and Clinical Epidemiology (CISPEC), Faculty of Health Sciences Eugenio Espejo, Universidad UTE, Quito, Ecuador

**Keywords:** bioceramic, case report, endodontics, nonsurgical repair, perforation, tricalcium phosphate

## Abstract

Root canal perforations are frequent but critical complications in endodontic treatments, which can pose a risk of irreversible damage to the affected tooth. These perforations typically occur during root canal preparation or endodontic post placement, presenting significant therapeutic challenges. The main objective in the management of such complications is to seal the perforation, thus preventing bacterial invasion and tooth loss. This report details the case of a 70-year-old patient with a perforation in the palatal wall of the middle root section of tooth #11. The treatment approach involved magnification techniques and the application of a bioactive material known as Biodentine. This case underscores the critical role of accurate radiographic evaluation and the use of biocompatible materials in the management of root perforations. Despite inherent challenges, proper diagnosis and planning, combined with appropriate techniques and materials, can lead to satisfactory results, effectively preserving the structure of the tooth.

## 1. Introduction

Root perforation is a complication that can occur during endodontic, periodontal, and restorative procedures. It is defined as an artificial communication between the root canal and the periodontium, which can have serious consequences if not properly managed. Primary etiologies include improper use of rotary instruments, clinician inexperience, intricate root canal anatomy, and pathological root erosion [1]. Root perforations can occur inadvertently during endodontic treatment or during the preparation of a postspace for prosthodontic treatment. Reports indicate that such incidents occur in approximately 3% to more than 10% of cases [1, 2]. Therefore, prompt detection, localization, and immediate repair of these perforations are crucial to ensure satisfactory long-term outcomes [1]. Furthermore, this type of accident is considered to be a factor of endodontic failure [3].

Teeth with a higher incidence of root perforations include the maxillary lateral incisor, 18.75%, and the first mandibular first molar, with 16.25% [4]. Regarding the location of the perforations relative to the third root, studies have shown that in the third coronal, the most affected teeth were the upper first premolar (10.0%) and the upper lateral incisor (8.75%), followed by the lower first premolar (3.75%) and the lower lateral incisor (3.75%). In the middle third, the upper lateral incisor was the most compromised (10%) and in the apical third, the lower first premolar was more affected (2.50%) than other upper and lower teeth [2]. In 60% of cases, perforations are due to endodontic treatment, while in 40% of cases, they are due to preparation and/or post placement [1].

The early diagnosis of root perforation is essential for successful treatment and is based heavily on a detailed clinical and radiographic evaluation. The treatment of root perforation is aimed at sealing communication to prevent the spread of bacteria and loss of periodontal tissue. Treatment modalities vary depending on the location, size, and cause of the perforation and may include nonsurgical and surgical techniques [4]. It is important to emphasize that prevention of root perforation is fundamental and can be achieved through careful planning of dental procedures and the appropriate selection of materials and techniques. Dental professionals should be well trained to manage root perforation effectively and safely [1, 4].

This case report describes the sealing of root perforations in the middle third of the maxillary central incisors (#11 and #21) using Biodentine (Septodont, Saint-Maur-des-Fossès, France), a bioactive material based on tricalcium silicate.

## 2. Case Report

A 70-year-old male patient was referred to our clinic from the undergraduate clinic of a university, with a dental record that indicated the beginning of endodontic treatment of tooth #11. The record noted that had pulp canal obliteration, with a previous diagnosis of pulp necrosis and a healthy periodontium. During the opening of the access, a deviation was observed in the canal at the middle third, leading the patient being directed to the postgraduate clinic of a university due to the limited resolution capacity at the initial facility.

**Figure 1:**
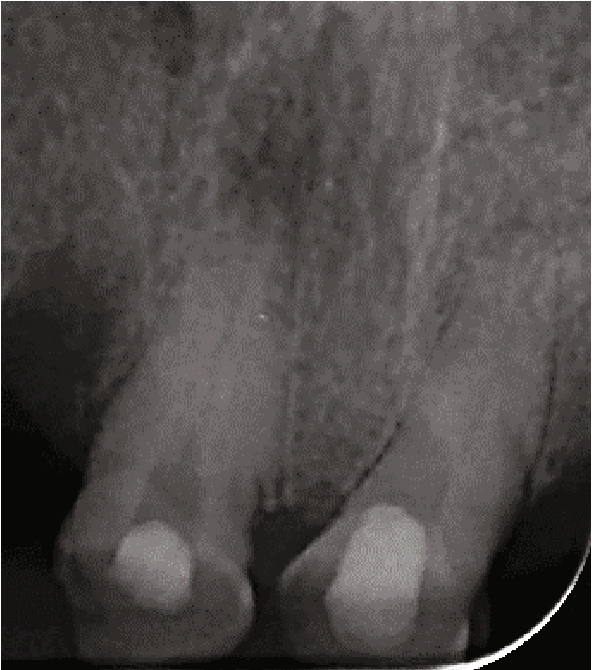
Initial radiograph of teeth #11 and #21.

When reviewing the patient's medical history and performing a clinical evaluation, we observed partial edentulism, loss of vertical dimension, and the presence of seven teeth showing attrition, all of which require endodontic treatment for prosthetic purposes. Periapical radiographs of existing teeth revealed, in most cases, canal obliteration likely due to dental attrition. The patient expressed a desire to prioritize treatment for teeth #11 and #21. Radiographic analysis revealed the following:

− Tooth #11: a coronal radio-opaque shadow indicative of restorative material and a radiolucent shadow suggesting a potential deviation in chamber access (Figure 1). Clinically, there were no signs of intraoral or extraoral inflammation. Percussion and palpation tests were nonreactive, and periodontal examination was stable with no mobility of the teeth. The diagnosis was a previously initiated tooth with a healthy periapex.
− Tooth #21: a slight radiolucent shadow indicating a root canal that begins nearly in the third cervical. Sensitivity tests showed reduced responses to cold and heat. Percussion and palpation tests, periodontal probing, and tooth mobility were normal. The diagnosis was a healthy pulp and periodontium.

**Figure 2:**
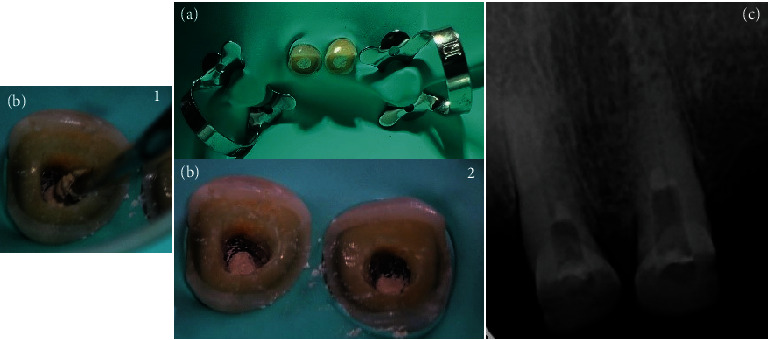
(a) Isolation of both teeth with rubber dam; ((b) 1 and 2) perforation sealing; and (c) postoperative radiograph with perforation sealing.

Treatment of teeth #11 and #21 began with the administration of 2% lidocaine with epinephrine 1:100,000 for effective anesthesia, followed by careful isolation of both teeth with rubber dam (Figure 2(a)). In the case of tooth #11, after the removal of the existing filling material, a perforation was clinically discerned with the help of a dental operating microscope (C-CLEARE-1, Coxo, China) using magnification levels of 8x and 12x. This finding was further corroborated by an apical locator and confirmed by periapical radiography, ensuring precision of diagnosis.

**Figure 3:**
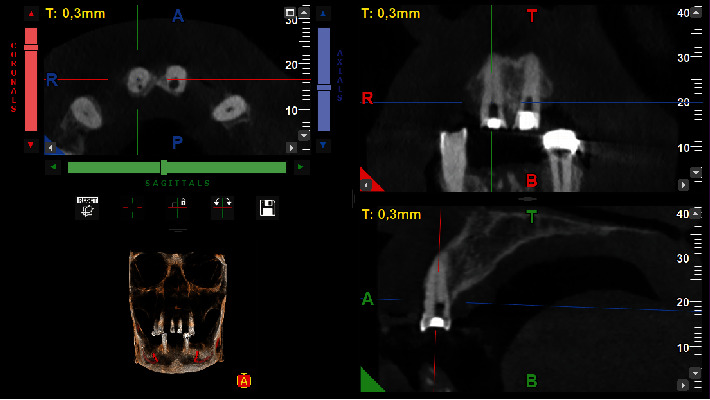
Tomographic slices of tooth #11.

**Figure 4:**
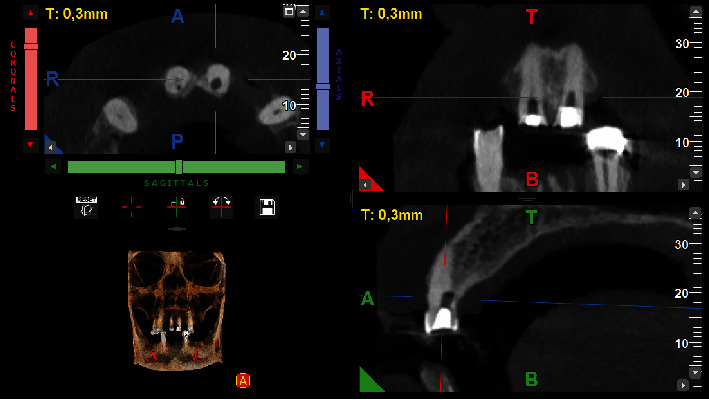
Tomographic slices of tooth #21.

The perforation site was thoroughly irrigated with saline to clean the area, followed by the application of a cotton pellet for temporary sealing. In addition, camphorated paramonochlorophenol was used as an intracanal drug. This choice was prescribed by the fact that the root canal had not been instrumented at this stage and it was imperative to mitigate any potential microbial contamination before proceeding. Therefore, the endodontic treatment was postponed until the following day, with a temporary filling placed to secure the tooth in the meantime. During the same time, the treatment started on tooth #21. Due to the tilt of the tooth and the obliteration of the pulp chamber, an accidental palatal perforation occurred during opening of the access. The same protocol as tooth #11 and the patient was informed of the incident. A cone beam computed tomography (CBCT) scan was requested to accurately assess the extent and location of the perforation. The scan was performed with a field of view of 5 × 5 cm and high-resolution settings, using I-MAX 3D PRO (Owandy, France). Based on the imaging results, plans were made to seal the perforations and continue treatment at the next appointment (Figures 1, 3, and 4).

During the first appointment to seal the perforations, a protocol involving 2% lidocaine with epinephrine 1:100,000 for anesthesia and absolute isolation was followed (Figure 2(a)). The removal of temporary filling material preceded the location of the canals and perforations in the middle third to the palatal side using a microscope (C-CLEARE-1, Coxo, China) at magnifications of 8x and 12x. The cavity was cleaned and disinfected, preparing it for the application of bioactive materials. Biodentine was used to seal the perforations, following a specific manipulation protocol. Initially, the powder capsule was placed in the amalgamator for 8–10 s to disperse and unify the powder. Then, five drops of liquid were added to the capsule and the amalgamator was set for 30 s at 4000–4200 rpm. A dense/malleable consistency was achieved, and the material was applied to the cavity using Condensers 3 and 4 with gentle pressure, ensuring complete sealing of the perforations while keeping the canal entrances clear (Figure 2(b), 1 and 2). The setting times of the material were carefully monitored with an initial setting time of 6 min and a full setting time of 12 min. Due to time constraints and the patient's fatigue, it was decided to postpone root canal preparation until a later appointment. Meanwhile, the teeth were temporarily sealed with sterile Teflon and a light-cured glass ionomer (Figure 2(c)).

**Figure 5:**
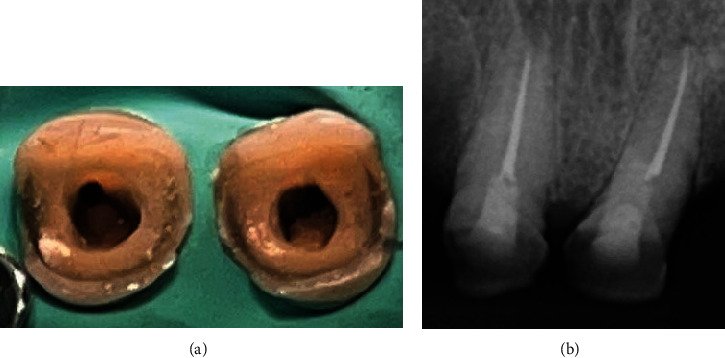
(a) Instrumentation of root canals completed; (b) final postoperative radiograph.

At the second appointment for instrumentation and obturation, the patient was asymptomatic, and treatment was continued. The previously established anesthesia protocol was once again used, using infiltrative techniques both buccal and palatal. Absolute isolation and removal of temporary material were performed, followed by irrigation with 5.25% NaOCl. The determination of the working length was achieved accurately with the use of an apical locator, with radiographic confirmation to validate the precision of this measurement. Mechanical instrumentation was carried out using the Race EVO system up to the 30/04 apical file, and the primary gutta-percha cone for the obturation was carried out with the primary 30/04 cones. A specific irrigation protocol was followed, and the canals were obturated using a single-cone technique, using Cerafill RCS bioceramic sealant (PREVEST DenPro, Jammu Kashmir, India) to ensure a hermetic seal. Finally, the canal entrances were sealed with flowable resin and the cavities were temporarily filled with resin for later rehabilitation (Figure 5).

**Figure 6:**
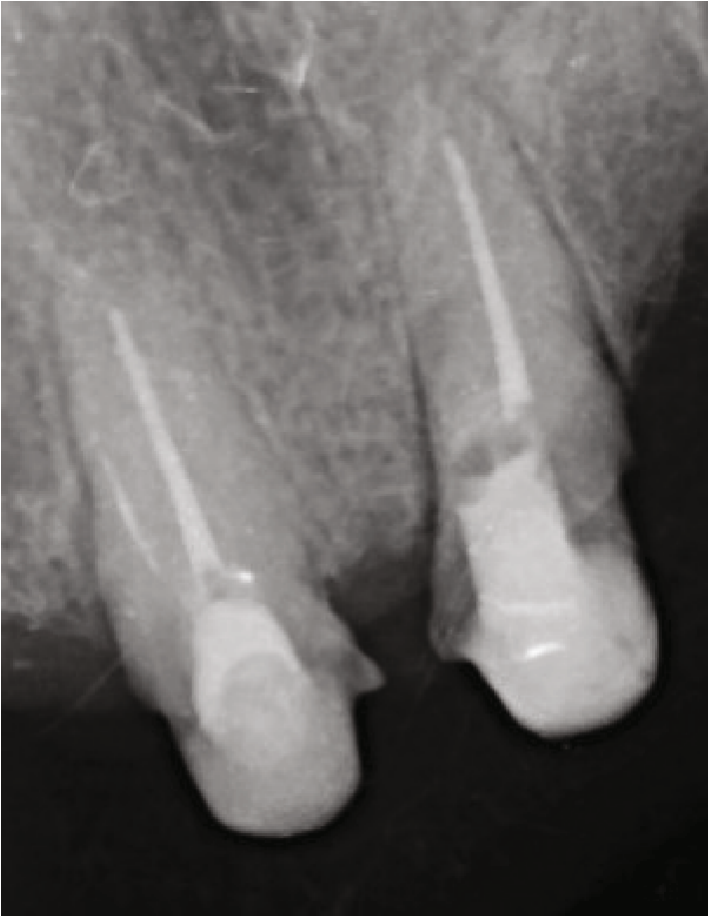
Twelve-month radiographic follow-up.

Periodic follow-up examinations for 12 months showed that the patient remained asymptomatic with no radiographic changes, indicating the successful outcome of the treatment. The rehabilitation phase culminated in the fitting of zirconium crowns, chosen for their aesthetic and functional properties, which effectively restored the patient's dental health and appearance.  Figure 6 shows the follow-up radiograph taken at 12 months.

**Figure 7:**
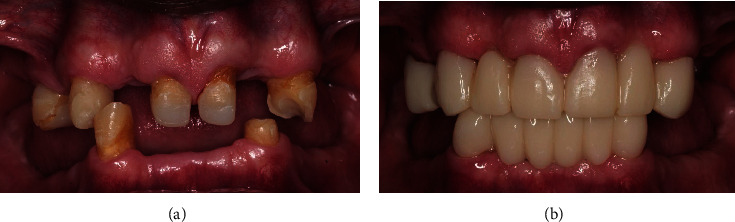
(a) Pretreatment intraoral photograph—frontal view; (b) posttreatment intraoral photograph—frontal view.

From the patient's point of view, the patient was very satisfied with the outcome of the treatment, highlighting the relief and happiness he felt when he was able to keep the affected tooth. This positive feeling extended to the posttreatment phase, where the patient expressed a higher level of satisfaction with the appearance of his teeth after rehabilitation and fitting of zirconia crowns (Figure 7).

## 3. Ethical Considerations

This case report was conducted after the patient signed the informed consent form and agreed to the treatment plan. Furthermore, the patient consented to the use of clinical information and photographs for anonymous presentation for publication in a scientific journal.

## 4. Discussion

This clinical case highlights the importance of a rapid and accurate approach to the management of root perforations. Early intervention with a biocompatible material such as Biodentine, characterized by its adequate sealing capacity, compression and flexion strength, and shorter set time, proved to be an effective option in the treatment of middle-third root perforations, as demonstrated in this case. The choice of material and technique used was based on a careful evaluation of factors influencing the success of treatment, such as the location and size of the perforation, the absence of apical infection, and repair in the maxillary teeth. Furthermore, the use of magnification techniques during treatment allowed greater precision in the detection and sealing of the perforation, which is essential to ensure the long-term success of treatment. This case also highlights the need to consider the dental anatomy and individual circumstances of each patient to optimize clinical outcomes.

To ensure the long-term success of a perforated tooth, immediate repair using a biocompatible material is crucial. This approach prevents bacterial contamination and restores the health of the periodontal ligament. In this case, the perforations were nonsurgically sealed the day after the incident, suggesting a higher success rate. According to studies by Siew, Lee, and Cheung root perforations treated in this manner exhibit a 72.5% success rate [1]. Additional factors that could ensure a favorable outcome in this case include the size of the perforations (1 mm) and their location in the middle third. Estrela et al. study indicate that these factors impact the prognosis, as they provide better visualization of the perforation and easier access to seal compared to a perforation located in the apical third [2].

A perforation near the gingival sulcus is more prone to bacterial contamination and therefore has a lower survival capacity. In contrast, a perforation in the middle third, as in the case presented, responds better due to its location. The challenge of sealing the perforation also varies depending on the level at which it occurred. Other factors that influence success include the anatomical location of the tooth in the dental arch [5], the presence of infection at the time of occurrence, the operator's expertise, the type of restoration performed, and the use of magnification devices during treatment. Magnification improves the detection of the precise perforation site and ensures proper sealing that is not visible to the naked eye [2].

Studies suggest that upper jaw tooth repair and the absence of apical infection correlate with a better success rate [4, 6]. These factors are also present in the case discussed, suggesting an improved long-term prognosis [6]. According to Sinkar et al., the repair of the maxillary teeth is more successful than the repair of the mandibular teeth [4]. The presence of infection at the perforation site during treatment adversely affects the outcome. Furthermore, Parirokh et al. found that preoperative radiolucency at the perforation site also negatively impacted treatment prognosis [7].

The term “bioactive endodontic material” refers to substances that, regardless of their chemical composition, can elicit a biological response in the surrounding tissue [8]. These materials release calcium and stimulate the production of apatite crystals, the formation of bone, dentin, or cementum, and the regeneration of the periodontal ligament. Calcium silicate cements are bioactive and hydrophilic and can be established in the presence of biological fluids, though their adhesive strength may be affected by these fluids. There is a wide variety of calcium silicate dental materials available, which complicates the choice for a specific case [5]. On the other hand, tooth discoloration after regenerative endodontic procedures affects the quality of life, especially in anterior teeth. Adequate knowledge of discoloration factors is crucial for successful endodontic therapy [9]. Some studies have investigated the color stability of mineral trioxide aggregate (MTA) and Biodentine and found that exposure to blood exacerbates discoloration more in MTA due to its longer setting time, whereas the faster setting time of Biodentine limits blood absorption and improves color stability [10].

It is essential that the material used to repair the perforations has excellent marginal sealing properties to prevent bacterial infiltration. The superior sealing ability of MTA has been attributed to the small particle size of its components, which increases the surface area available for powder hydration and allows penetration into the dentinal tubules [1]. This is also related to a slight expansion during the setting process and the production of crystalline hydroxyapatite at the interface of the material and dentin walls. Studies that compare the sealing capacity, microfiltration, and marginal adaptation of Biodentine and MTA have produced controversial results. Some studies indicate that MTA has more favorable sealing and marginal adaptation properties than Biodentine when used as a filling material for the repair of furcal perforations. However, others suggest that the marginal adaptation and sealing capacity of Biodentine surpass those of MTA [11, 12]. Aggarwal et al. noted that a favorable characteristic of Biodentine is that blood contamination does not affect its bonding strength, regardless of the setting time [13]. Subramanyam and Vasantharajan found that the presence of blood or saliva does not affect the compressive strengths of MTA or Biodentine [14]. However, other authors assessed the influence of blood contamination on the push-out bond strength of three bioactive materials on root canal dentin and stated that ensuring a proper seal is crucial for root canal filling materials [15]. Calcium silicate-based cements are often exposed to blood contamination in various clinical applications, and adequate bond strength at the biomaterial–root canal dentin interface is essential to resist detachment and resist masticatory forces [16].

Although MTA has a high efficacy rate of 80.9%, its long setting time is a reason for its impracticality in clinical procedures requiring single-session completion [7]. Biodentine, on the other hand, sets faster than other materials and has greater compressive and flexural strength than MTA, approaching dentin, making it the preferred choice for sealing perforations in this case [11, 12]. However, studies have shown that these calcium silicate-based cements, such as MTA and Biodentine, exhibit color changes when exposed to blood in the medium and long term [10, 17]. The discoloration is more pronounced in MTA than in Biodentine [17, 18]. Biodentine, with its shorter setting time, improved handling properties, and reported ability to stimulate mineralized tissue formation, may offer advantages over traditional MTA in the treatment of middle-third root perforations. Similarly, the bioceramic-based TotalFill has shown promising results in terms of sealing ability and biocompatibility, making it a viable alternative to consider [5, 19, 20]; however, clinical trials are needed to determine the clinical effect of this material on the long-term outcome of endodontic treatment.

Preventing complications such as root perforation is fundamental and requires careful planning and technique during endodontic procedures. However, when they do occur, the selection of the appropriate material and the implementation of a treatment protocol based on scientific evidence are essential for the success of treatment. This case reinforces the idea that with appropriate material selection and efficient clinical management, even complex clinical situations can be successfully resolved, thus preserving the integrity and function of the tooth in the long term.

## Data Availability

Data sharing is not applicable to this article, as no datasets were generated or analyzed during the current study.
